# An Efficient Multi-Scale Convolutional Neural Network Based Multi-Class Brain MRI Classification for SaMD

**DOI:** 10.3390/tomography8040161

**Published:** 2022-07-26

**Authors:** Syed Ali Yazdan, Rashid Ahmad, Naeem Iqbal, Atif Rizwan, Anam Nawaz Khan, Do-Hyeun Kim

**Affiliations:** 1 Department of Computer Science, Attock Campus, COMSATS University Islamabad, Attock 43600, Pakistan; aliyazdan.myworld@gmail.com; 2 Big Data Research Center, Jeju National University, Jeju 63243, Korea; 3 Department of Computer Engineering, Jeju National University, Jeju 63243, Korea; naeemiqbal@jejunu.ac.kr (N.I.); atifrizwan@jejunu.ac.kr (A.R.); anamnawaz@jejunu.ac.kr (A.N.K.); 4 Advanced Technology Research Institute, Jeju National University, Jeju 63243, Korea

**Keywords:** MRI, brain tumor, deep learning, multi-scale convolutional neural network, classification, FSNLM

## Abstract

A brain tumor is the growth of abnormal cells in certain brain tissues with a high mortality rate; therefore, it requires high precision in diagnosis, as a minor human judgment can eventually cause severe consequences. Magnetic Resonance Image (MRI) serves as a non-invasive tool to detect the presence of a tumor. However, Rician noise is inevitably instilled during the image acquisition process, which leads to poor observation and interferes with the treatment. Computer-Aided Diagnosis (CAD) systems can perform early diagnosis of the disease, potentially increasing the chances of survival, and lessening the need for an expert to analyze the MRIs. Convolutional Neural Networks (CNN) have proven to be very effective in tumor detection in brain MRIs. There have been multiple studies dedicated to brain tumor classification; however, these techniques lack the evaluation of the impact of the Rician noise on state-of-the-art deep learning techniques and the consideration of the scaling impact on the performance of the deep learning as the size and location of tumors vary from image to image with irregular shape and boundaries. Moreover, transfer learning-based pre-trained models such as AlexNet and ResNet have been used for brain tumor detection. However, these architectures have many trainable parameters and hence have a high computational cost. This study proposes a two-fold solution: (a) Multi-Scale CNN (MSCNN) architecture to develop a robust classification model for brain tumor diagnosis, and (b) minimizing the impact of Rician noise on the performance of the MSCNN. The proposed model is a multi-class classification solution that classifies MRIs into glioma, meningioma, pituitary, and non-tumor. The core objective is to develop a robust model for enhancing the performance of the existing tumor detection systems in terms of accuracy and efficiency. Furthermore, MRIs are denoised using a Fuzzy Similarity-based Non-Local Means (FSNLM) filter to improve the classification results. Different evaluation metrics are employed, such as accuracy, precision, recall, specificity, and F1-score, to evaluate and compare the performance of the proposed multi-scale CNN and other state-of-the-art techniques, such as AlexNet and ResNet. In addition, trainable and non-trainable parameters of the proposed model and the existing techniques are also compared to evaluate the computational efficiency. The experimental results show that the proposed multi-scale CNN model outperforms AlexNet and ResNet in terms of accuracy and efficiency at a lower computational cost. Based on experimental results, it is found that our proposed MCNN2 achieved accuracy and F1-score of 91.2% and 91%, respectively, which is significantly higher than the existing AlexNet and ResNet techniques. Moreover, our findings suggest that the proposed model is more effective and efficient in facilitating clinical research and practice for MRI classification.

## 1. Introduction

Brain tumors are masses formed due to the abnormal growth of lesions inside human brains that directly affect the functionality of brains for controlling voluntary and involuntary processes [1]. It is a life-threatening and leading disease toward cancer mortality worldwide [2]. According to data from the International Agency for Research on Cancer 2012, brain tumors are the 22nd most common form of tumor. However, it is 12th in terms of the mortality rate [3]. According to Cancer Statistics 2019, there has been an increase in deaths caused by different types of cancer, including brain cancer [4]. A report published by World Health Organization (WHO) estimated that 9.6 million fatalities were caused by diagnosed cancer around the globe in 2019 [5]. In [6], 296,851 new cases of brain tumors and other spinal tumors were reported worldwide in 2018. In 2017, among all types of tumors, brain and other spinal cord tumors were the leading cause of death in men below 40 and women below 20 years of age [7]. Furthermore, the survival rate of the patient decreases with age. The survival rate for adults above 40 is about 21 percent [8].

Neuroimaging is a powerful non-invasive tool for finding abnormalities in the brain. Computerized Tomography (CT) scans and MRIs are the two most commonly used neuroimaging techniques for brain tumor diagnosis [9,10]. The advantage of using MRI over a CT scan in brain imaging is that not only does it provide a better tissue contrast, but it also does so without the use of radiation [11]. MRI uses strong magnetic fields and radio waves to analyze the anatomy and physiological processes of a body. Therefore, MRI is a strong tool for detecting diseases and anatomical anomalies in a body. It has a wide range of applications in medical imaging. For example, cardiovascular MRI is used for structural analysis of the heart. Musculoskeletal MRI is used to assess the spine, joint diseases, and soft tissue tumors. Similarly, brain MRI is used to detect neurological diseases such as tumors, clots, etc. [12]. However, detecting a tumor in a brain MRI is difficult and requires an expert to examine the images to determine the presence of a tumor. It is also imperative to detect and identify the type of tumor. Therefore, Machine Learning (ML) has become a widely used paradigm for detecting tumors in several body parts, including brain tumor detection in MRI [13], liver tumor classification [14], breast tumor detection [15], etc. Hence, a robust solution is needed to utilize a Computer Vision (CV) technology that can accurately detect and identify the presence of a tumor in an MRI.

MRIs suffer from the inherited problem of being vulnerable to noise [16,17]. Getting the desired image resolution decreases the Signal to Noise Ratio (SNR) [18]. Noise can occur in any listed processing phase: acquisition, compression, pre-processing, storage, transmission, and/or reproduction. The transformation of MRI images into magnitude images changes the Gaussian distribution into a Rician distribution [19]. The presence of noise in the MRIs makes it difficult to perform any further image processing techniques on these images [20]. Therefore, there is a need for noise removal as pre-processing to pass the pre-processed images to the ML classifier for accurate tumor detection. Several techniques have been used to denoise the MRIs [21]. However, denoising filters also affect the level of detail in the images, which affects the classification process. Hence, FSNLM [22] is employed to perform noise removal, but it has a comparatively low processing cost. This technique uses fuzzy logic to find segments in the image that are similar to the noisy pixel and use these segments to find the noise-less pixel.

ML techniques have been proven promising for identifying and classifying a brain tumor in MRI images [23]. Therefore, developing an accurate classification model to detect a brain tumor in MRI images is quite possible. However, conventional ML techniques: Support Vector Machine (SVM) [24], k-Nearest Neighbor (KNN) [25], Decision Tree (DT) [26], Random Forest (RF) [27], etc., require a lot of knowledge of the input images so that useful features can be extracted from them. Deep Learning (DL) techniques have recently revolutionized ML by automatically extracting features from MRI images [28]. Convolutional Neural Networks (CNNs) are widely used DL techniques that perform automatic feature extraction, making them suitable for image classification and object detection problems [29,30]. A CNN performs a series of convolutional, non-linearity, and pooling operations on the input images to extract the useful features from given images. These features are then used for object detection, segmentation, or image classification. Moreover, the traditional CNN model requires a huge amount of training samples to produce effective classification results. Therefore, our research study focuses on transfer learning that allows pre-training a CNN model on a vast image dataset and then reusing the resulting parameters of a model for a similar task with specific samples.

The transfer learning approach is very effective for training a DL architecture from scratch when training samples are small, causing a biased and over-fitted DL model. Several pre-trained models, such as GoogleNet, AlexNet, ResNet, and VGG, were originally designed for different brain tumor classification tasks [31]. However, these architectures have a large number of trainable parameters and hence require a lot of computational resources. Furthermore, the size of the convolution kernel governs the features extracted by the CNN. Using a single kernel restricts the extractions of a wider range of features from the input images. Therefore, utilizing more than one kernel for a single convolution is imperative. Hence, our study proposes a multi-scale CNN model to enhance the performance and efficiency of traditional CNN architectures.

The notable contributions of the proposed research study are as follows:

- The impact of convolutional scaling is evaluated for the varying tumor size, shape, and location on the classification performance of CNN.
- An empirical scale-based multi-scale CNN architecture is proposed to outperform the state-of-the-art CNN architectures in terms of effectiveness and efficiency.
- The proposed multi-scale CNN model is tested on noisy and denoised images, concluding that noise affects the CNN classifier accuracy.
- Furthermore, a detailed comparative analysis is presented to evaluate the accuracy and efficiency of the proposed research study.

The remaining structure of the proposed study is ordered as follows: Section 2 describes the theoretical background of the computer-aided diagnosis of brain tumor MRIs. Section 3 discusses the proposed model for brain tumor detection. Section 4 consists of the experimental environment, experimental results, and performance analysis. Section 5 presents a comparative analysis to evaluate the accuracy and efficiency of proposed and existing approaches. Section 6 concludes the proposed research study, and possible future directions are also suggested for research in this field.

## 2. Related Work

This section presents existing studies related to brain tumors and the general methods used to diagnose them. It also discusses the various ML techniques that have been used to develop Computer-Aided Diagnosis (CAD) systems for brain tumors. Furthermore, different CNN architectures are discussed for brain tumor detection, classification, and segmentation.

### 2.1. Brain Tumor

The brain is undoubtedly an essential organ of the human body. It consists of a complex mechanism in which a network of billions of neurons controls all the body’s functions. The human body creates billions of cells per day. A cell will eventually die after completing its life cycle and be replaced by a new one in a normal situation. However, sometimes this cycle is disrupted. Some cells do not die; instead, they continue to grow in an uncontrolled manner [32]. A group of such cells forms a tumor. A tumor can form in any part of the body. A tumor in the brain is called a brain tumor [33]. The brain is enclosed inside a rigid skull. Tumor growth in such a restricted area can press against the brain. This can not only cause brain damage but possibly be fatal.

Brain tumors can be classified into different types based on their origin, aggressiveness, and location. A brain tumor may be primary or secondary. Primary brain tumors originate in the brain, while secondary brain tumors originate in another part of the body, such as the lungs, and then metastasize to the brain. Based on the nature and aggressiveness of their growth, tumors have three categories: benign, pre-malignant, and malignant [34]. A benign tumor is not cancerous, meaning it will not metastasize to other body parts. A pre-malignant tumor is not yet cancerous but risks becoming cancerous. However, a malignant tumor is cancerous. It grows over time in size and can even metastasize to other organs. Malignant tumors can eventually become fatal. Therefore, they must be surgically removed and possibly treated with chemotherapy, radiation, or immunotherapy. In terms of location [35], brain tumors have different types: glioma, pituitary, and meningioma. Glioma is cancer formed in the central or peripheral nervous system due to glial cells. Two-thirds of all primary cancerous tumors are categorized as glioma [36]. Three layers of membrane called meninges cover the brain and the spinal cord. A benign tumor formed in the interior of the pituitary gland is called a pituitary tumor. The pituitary gland is a small gland responsible for secreting hormones vital for regulating body functions. Pituitary tumors account for 8% of all primary brain tumors [37]. Meningioma is one of the most common forms of brain tumor that form in the meninges. Out of all the cases of brain tumors, nearly 20 percent are meningioma. Meningioma can be malignant but are mostly (90%) benign. They either grow at a very slow rate or do not grow at all. In most cases, they do not require any immediate treatment [38]. However, it is important to detect the presence of a tumor in neuroimaging and identify the type of tumor detected.

Furthermore, CT scan and MRI are the two main neuroimaging techniques for brain tumor diagnosis without any invasive method. MRI is preferable to a CT scan for two main reasons: it does not use radiation and provides better contrast than the CT scan [39]. A brain tumor diagnosis is initially conducted using MRI. Once the presence of a tumor has been confirmed, other tests such as tissue analysis can be conducted to get more details about the tumor [40].

### 2.2. Noise Removal from MRIs

Noise in MRIs makes it challenging to apply image processing techniques to detect brain tumors. It also negatively influences the classification accuracy of the brain tumor detection systems [41]. Therefore, different noise removal filters have been developed to enhance the quality of MRIs for diagnosis. In [42], the authors proposed a denoising technique for MRIs to perform classification for brain tumor detection. The technique was developed by combining Low-Rank Matrix Decomposition (LRMD) and SVM. An adaptive hexagonal fuzzy hybrid filter proposed by Kala et al. [43] removes noise from MRI images using local and non-local filters to improve SNR. Zeng et al. [44] proposed a denoising technique for MRIs. Their method consisted of three stages. In the first stage, an MRI image is segmented into cartoon, texture, and residual parts using Morphological Component Analysis (MCA). In the second stage, the three segments are denoised using the wiener filter, hard wavelet threshold, and soft wavelet threshold. In the third stage, all the denoised segments are combined for a denoised MRI. Dictionary learning-based algorithms have proven to be quite effective for noise removal while preserving necessary details, but at a very high computational cost [45]. However, noise removal filters affect MRI information (pixels) that negatively affect the brain tumor classification processes. Therefore, it is required to preserve MRI information while performing the denoising process. Hence, our research study utilizes the FSNLM filter [22] to remove noise from MRIs at a low computational cost compared to other noise removal filters.

### 2.3. Traditional CADs for Brain Tumor

There is a need for a CAD system to classify MR images into tumor and non-tumor. A lot of work has been performed in brain tumor detection and classification. In [46], the authors designed a model for brain tumor MRI classification based on offline and online phases. In the offline phase, MRIs are processed in a sequence of steps: tumor segmentation, feature extraction, and distance metric learning. The online phase processes MRIs to extract features and compare them with the learned distanced metrics stored in the online database. In [47], the authors proposed a hybrid technique based on Fuzzy C-mean clustering and multi-object optimization for brain tumor tissue segmentation. A study presented in [48] performed segmentation on MRI images using the Skippy greedy snake algorithm. In [49], the authors used watershed and thresholding-based techniques for tumor segmentation. Similarly, a threshold-based tumor detection technique was used in [50]. Another detailed review study was presented in [51] for different brain tumor MRI segmentation techniques. The four techniques studied were K-Mean, Fuzzy C-mean, region growing, and Otsu segmentation. Based on the results, it was concluded that K-mean outperformed other segmentation techniques. In [52], the authors used Fuzzy C-mean clustering for tumor detection in MRIs. Furthermore, a study presented in [53] used SVM-based techniques for tumor classification. The presented study consisted of four steps. First, the region of interest was manually extracted from the images. Second, the tumor was segmented from the images using thresholding. Third, a Genetic Algorithm (GA) extracted features from the images. Finally, an SVM with a linear kernel function was used to classify the images.

### 2.4. Traditional CNN Architecture

In ML, DL is an emerging and prominent research topic used in different domains. CNNs are a type of Deep Neural Network (DNN) mostly used in computer vision problems. Traditional supervised learning techniques required hand-crafted feature extractors. The quality of the model depended on how accurately these feature extractors suit the input data. Designing these extractors is time-consuming and requires a lot of prior knowledge about the input data. Another disadvantage of these designed feature extractors is that they seldomly reflect real-world data [54]. The core advantage of the CNN model is to extract the significant features without human supervision [55]. Therefore, it enables the automatic representation of promising features compared to the conventional ML. It also eliminates the need for manual feature extraction as pre-processing and enables us to work on large and unstructured datasets. This automatic feature extraction in CNN makes it preferable over other ML techniques in image segmentation, classification, and object recognition tasks [56].

### 2.5. CNN in CAD Systems

Due to the automatic representation of promising features, CNN has become popular in image classification, segmentation, and object detection problems. Therefore, it has been widely used in CADs for several diseases such as skin cancer, breast cancer, and lung cancer [57]. In [58], the authors used a simple CNN architecture for successfully detecting skin cancer in images. In [59], the authors achieved 98.94% accuracy in detecting breast cancer from mammogram images. Similarly, a lung cancer detection system was designed to detect lung cancer in low-dose computer tomography (LDCT) scans with an AUC of 0.913 [60]. In [61], the authors used a simple CNN for detecting Alzheimer’s disease in brain MRIs.

Similarly, CNN has also been very effective for brain tumor detection, classification, and segmentation [62,63,64]. In [62], a CNN was developed based on clustering in MRI images. In [63], the authors used a CNN for MRI image segmentation while using a small 3 × 3 kernel. In [65], the authors employed an Artificial Neural Network (ANN) in MR image classification. A study presented in [66] performed classification on MRI images for tumor detection using KNN, SVM, Linear Discriminant Analysis (LDA), and DNN and concluded that DNN outperformed the other classification techniques. In [67], the authors designed a simple CNN for brain tumor classification. They used three different types of input: original images, cropped images, and segmented regions. Gray Level Co-occurrence Matrix (GLCM) performed feature extraction, and then those features were used by CNN to perform classification [68]. Similarly, DNN was used with auto-encoders to classify brain tumors. The Discrete Wavelet Transform (DWT) and GLCM were used in the first phase to extract texture and intensity-based features from brain MRIs. These features were then passed on to a DNN, which consisted of two auto-encoders and one SoftMax layer to perform classification. Similarly, in [69], the authors used GLCM and DWT for feature extraction and then used a Probabilistic Neural Network (PNN) to classify the tumor images. In [70], the authors performed multi-class brain tumor classification on T1-weighted contrast-enhanced images on two different datasets using a simple CNN model. First, MRIs from the first dataset was classified into three tumor types: meningioma, glioma, and pituitary tumor. Second, the images from a different dataset were classified into different grades of glioma. In a study presented in [34], the authors performed tumor classification using CNN architectures with different depths. The proposed study was used to classify MRIs into three different tumor types: meningioma, glioma, and pituitary. The results concluded that increasing the depth of the architecture does not always improve accuracy. However, increasing the number of convolution filters does improve performance.

Some studies introduced randomized ensemble CNN for detecting brain tumors and early stages of Alzheimer’s disease. In [71], the authors suggested a novel 2D-CNN randomized ensemble approach to detect the early stages of Alzheimer’s disease using magnetoencephalography (MEG) activity. The proposed randomized ensemble approach handles both noisy and sparse data. Similarly, in [72], the authors developed a multi-path lightweight deep CNN model using randomized dilated convolutions. The proposed model consists of the two multi-path networks, where the features map of the first network is used as an input for the second network. The resulting analysis indicates that the proposed multi-path reduced the classification error by 0.8% compared to the conventional techniques.

### 2.6. Transfer Learning

Transfer learning has also shown great promise in medical applications. Transfer learning allows a CNN model trained for a separate related problem to solve the current problem. CNN models such as AlexNet [73], GoogleNet [74], and ResNet [75], which were originally trained on an ImageNet dataset consisting of 1000 different classes, can be retrained on a different dataset. The presented study in [76] performed a classification of CT images into benign and malignant renal tumors by using a pre-trained Inception V3 model. In [77], the authors used a pre-trained GoogleNet to classify MRIs into different types of tumors and achieved an accuracy of 98% and outperformed other state-of-the-art methods. Another study presented in [78] performed image classification using different pre-trained models: GoogleNet, ResNet, AlexNet, and SqeezeNet. While the AlexNet was able to give the highest classification accuracy, it had a very high computation cost compared to the other networks. In another research study [79], the authors used nine different pre-trained CNN models for brain tumor classification. The results indicated that VGG16, VGG19, and AlexNet perform better than deeper models such as ResNet, GoogleNet, and SeNEt.

### 2.7. Summary

To the best of our knowledge, all aforementioned studies attempted to utilize conventional ML and serial DL architectures to identify and classify tumors in brain MRIs and CT. However, the traditional ML techniques are ineffective because these models require extensive domain knowledge and time to manually extract features from the MRIs. In contrast, serial DL architectures utilized a uniform kernel size for the convolutional layers that restrict the feature extraction process. Furthermore, it is imperative to analyze small and large kernel sizes for extracting relevant features from brain MRI to enhance the performance of the brain tumor detection systems. Furthermore, the existing studies did not analyze the impact of magnetically injected Rician noise on classification results. Hence, our study is the first attempt to propose multi-scale CNN architectures with parallel convolution paths having different kernel sizes and analyze the impact of Rician noise in brain MRIs on the classification performance of CNN. Moreover, this study focuses on optimizing/shrinking trainable parameters of the proposed and existing DL architectures to enhance the efficiency of tumor detection in brain MRIs.

## 3. Proposed Methodology

**Figure 1:**
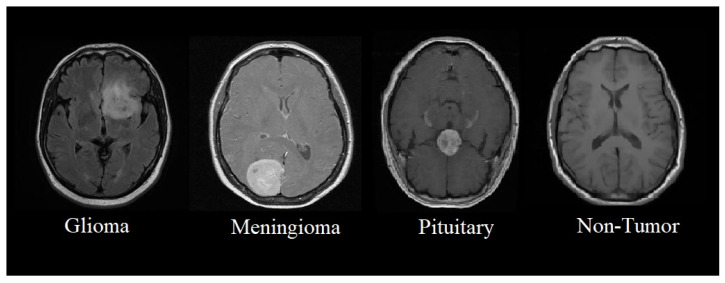
Classification of brain tumors from MRIs.

This section presents the proposed methodology of a multi-scale CNN model for identifying and classifying brain tumors in four classes such as glioma, meningioma, pituitary, and non-tumor MRI. The proposed multi-scale model aims to perform multi-class classification, where brain tumors are classified into four classes, as shown in Figure 1.

### 3.1. Overview of Proposed Model

**Figure 2:**
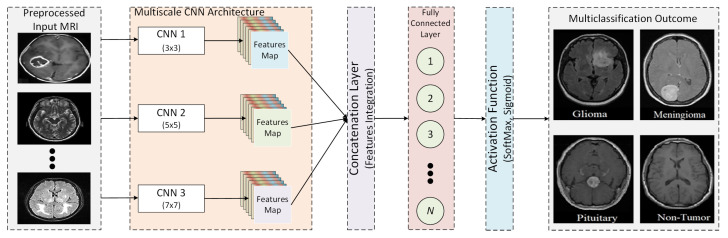
Block model of the proposed multi-scale CNN architecture.

An overview of the proposed multi-scale CNN model is presented in Figure 2. The proposed multi-scale CNN model consists of multiple parallel convolutions paths having different filter sizes. The main objective of the proposed multi-scale CNN architecture is to analyze the impact of convolutional filters having different sizes on the classification of brain tumors. Therefore, different filter sizes are considered, such as 3 × 3, 5 × 5, 7 × 7, and 9 × 9. First, the pre-processed MRI is passed to each convolution path to extract features. Each convolutional path has a different filter size with the same number of filters to extract features from pre-processed MRIs. Furthermore, each convolutional path consists of a uniform number of convolutional layers. In addition, the activation function and max pooling layers are used for each convolutional layer to map inputs into output and reduce computational complexity. Next, a concentration layer is deployed to integrate parallel convolutional path feature maps. The integrated features map is then passed to the fully connected layer, where each neuron of the fully connected layer is connected with neurons of the previous layer. Finally, a SoftMax method is used as an activation function that uses a probabilistic approach to predict the best class label for the given inputs. It is used to determine the probability for each class label to predict the best class label.

### 3.2. General Flow of Proposed Model

**Figure 3:**
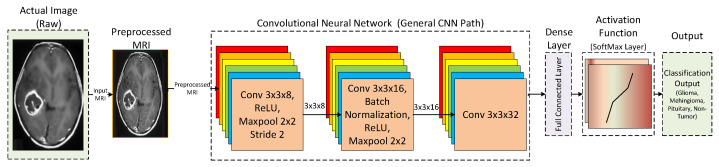
General architecture of a conventional CNN model.

In this subsection, a general flow of the CNN model is discussed. CNN architecture takes inspiration from the biological processes of a real eye. The pattern of connectivity among the neurons resembles that of the visual cortex of animals. A CNN performs several linear and nonlinear transformations on the input data to get more useful abstract representations [80]. In addition, CNN tries to learn the relationship between the pixels of the input images. The typical architecture of a CNN has an input layer, followed by alternating convolution and max pool layers. The output of these layers is then passed onto fully-connected hidden layers. Figure 3 shows the architecture of a generic CNN architecture.

#### 3.2.1. Convolutional Layers

The convolution layers consist of several convolutional filters that are used to extract features from the images. First, the input data is multiplied with a two-dimensional array of weights in this layer, called a kernel or a filter. Each kernel is designed to extract a specific type of feature. Then, the kernel is applied over the entire image. The result of this operation is a two-dimensional matrix called the feature map. Equation (1) gives the formula for calculating the feature map.

(1) $$G\left[m,n\right]=\sum_{j}\sum_{k}f\left[j,k\right]I\left[m-j,n-k\right]$$

where *G* is the output feature map, *I* is the input images, and *f* is the kernel. The network uses more than one filter to extract different types of features. Each filter gives a two-dimensional matrix as an output. The output matrices of all the filters are stacked to form a three-dimensional tensor. Equation (2) expresses the dimensions of the output tensor.

(2) $$\left(n,n,n_{c}\right)*\left(f,f,f_{c}\right)=\left[\left[\frac{n+2p-f}{s}+1\right],\left[\frac{n+2p-f}{s},n_{f}\right]\right]$$

where *n* represents the size of the images, *f* represents the size of the filter, $n_{c}$ indicates the number of channels, *p* indicates padding, *s* represents stride, and $n_{f}$ represents the total number of filters.

**Figure 4:**
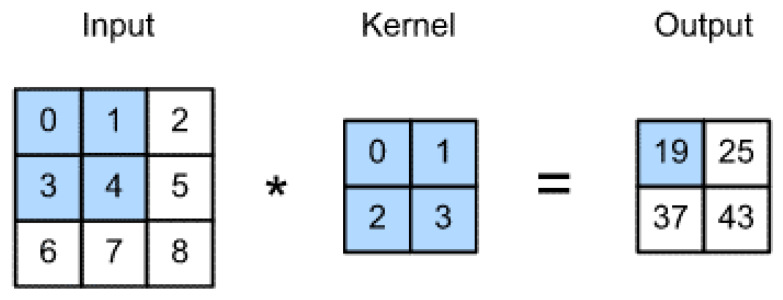
Convolutional example using a 2 × 2 filter.

These filters do not need to be designed by hand. Instead, the values of these filters can be learned during the training to determine what type of feature needs to be extracted. The first layer extracts simple, low-level features from the images. The later successor layers, however, extract more complex high-level features. Figure 4 describes a convolution operation.

#### 3.2.2. Non-Linearity Layer

The non-linearity layer is used to introduce non-linearity in the network. It uses a set of functions that are called the activation function. These functions are used to remove redundant data while preserving useful data [81]. The most commonly used activation function is the Rectified Linear Unit (ReLU) function [82]. Different variants of ReLU are available, such as Leaky ReLU (LReLU) [83], Parametric ReLU (PReLU) [84], and Randomized ReLU (RReLU) [85]. A simple description of the ReLU function is given in Equation (3).

(3) $$f\left(z_{x,y}\right)=\left\{\begin{array}{cc} z_{x,y}, & if\;z_{x,y}>0\\ 0, & if\;z_{x,y}\leq 0 \end{array}\right.$$

where $z_{x,y}$ is the input value at position (x,y).

#### 3.2.3. Pooling Layers

Pooling layers are used for down-sampling. They reduce the resolution of the output from the convolution layer. They combine a cluster of output neurons into a single neuron before sending the image to the next layer. Pooling can be max or average.

**Figure 5:**
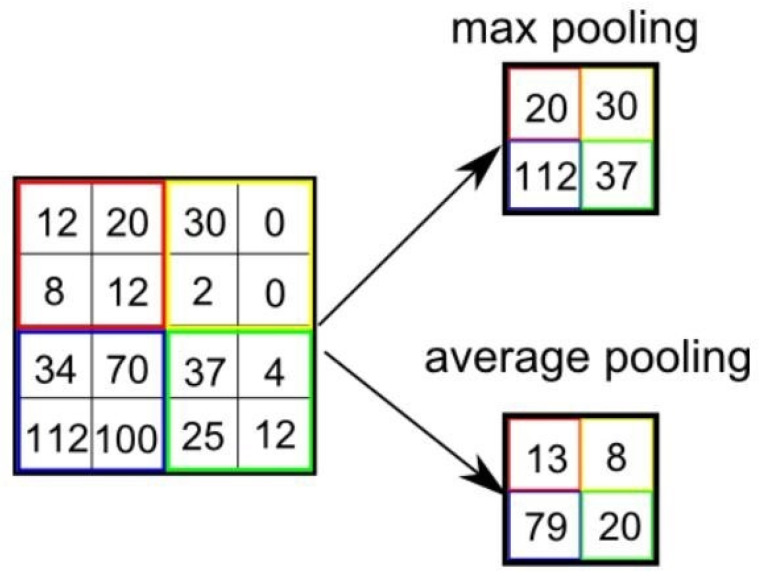
Comparison of max and average pooling.

Figure 5 describes the working of max pooling and average pooling operations using a 2 × 2 filter. The max pooling selects the maximum of the cluster, while the average pooling selects the cluster’s average. The purpose of pooling is to reduce the number of parameters and avoid over-fitting. The output size *M* of a pooling layer can be calculated by using Equation (4).

(4) $$m=\frac{n+2p-f}{s}+1$$

where *n* is the input image size, *f* is the size of the pooling filter, *p* is the padding, and *s* is stride.

Unlike the convolution layer, the pooling layer does not have any learnable parameters [86].

#### 3.2.4. Fully Connected Layer

**Figure 6:**
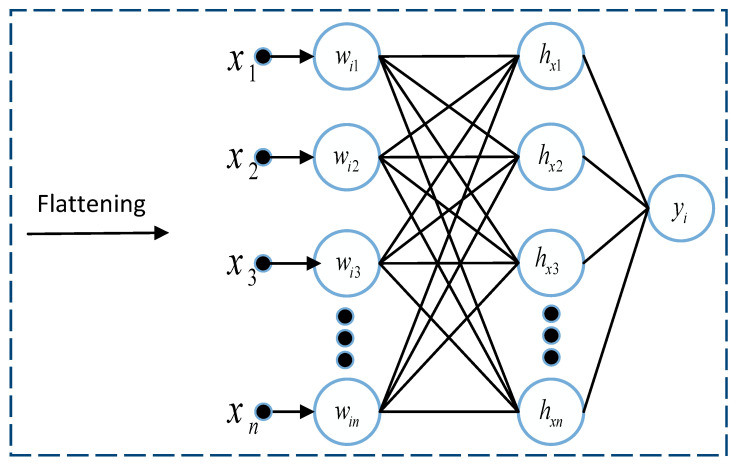
Basic structure of the fully connected layer.

The fully connected layer is like a Multi-layer perceptron. As the name suggests, in a fully connected layer, every single neuron in one layer is connected to each neuron in another layer. The 2D matrix from the previous layers is flattened and converted into a vector before passing to the fully-connected layer. This layer is usually used as an output layer. It uses the one-dimensional vector representation of the feature map of the image outputs the class label. Figure 6 describes the basic structure of the fully connected dense layer. It is the final layer where all the computations and reasoning are performed on the features received from the concatenation layer.

### 3.3. Proposed Multi-Scale CNN Architecture

The architecture of the proposed multi-scale CNN model is developed based on parallel convolutions paths. The architecture of the proposed multi-scale CNN model consists of the following main steps: a collection of MRI data, pre-processing of the acquired data, a configuration of multiple parallel convolutional paths, and performance analysis of multi-classification outcomes. First, MRI data are collected from Kaggle, consisting of 3264 images of four classes: glioma, meningioma, pituitary, and non-tumor images. Second, a pre-processing module is developed to pre-process raw MRI by removing noise to enhance the quality of the given MRIs. Third, a CNN model is developed based on multiple parallel convolutional paths to extract features from the pre-processed MRIs to detect and identify brain tumors. Finally, performance analyses are considered to evaluate the accuracy and efficiency of the proposed multi-scale CNN model. The detailed flow of the proposed multi-scale CNN model is as follows.

#### 3.3.1. Collection of MRI Data

In this research study, an open-source, publicly available MRI dataset is acquired from Kaggle [87]. The dataset contains 3264 MRIs from four classes: glioma, meningioma, pituitary, and non-tumor images and the combination of T1, T2, and Flair types. The acquired data consists of the commonly used MRI sequences, such as T1, T2, and Fluid Attenuated Inversion Recovery (Flair). T1 images are generated based on short Time to Echo (TE) and Repetition Time (RT). T1 properties of the tissue are used to determine the contrast and brightness of the MRI. Similarly, T2 images are generated using long TE and TR times. In T2 images, T2 tissue properties are used for contrast and brightness. Furthermore, the Flair sequence is similar to the T2 sequence but uses very long TE and TR times to produce the MRI. The class distribution of the dataset is given in Table 1. Out of 3264 MRIs, 926 MRIs belong to glioma, 937 to meningioma, 500 are from pituitary, and the remaining 901 are from non-tumor patients.

**Figure 7:**
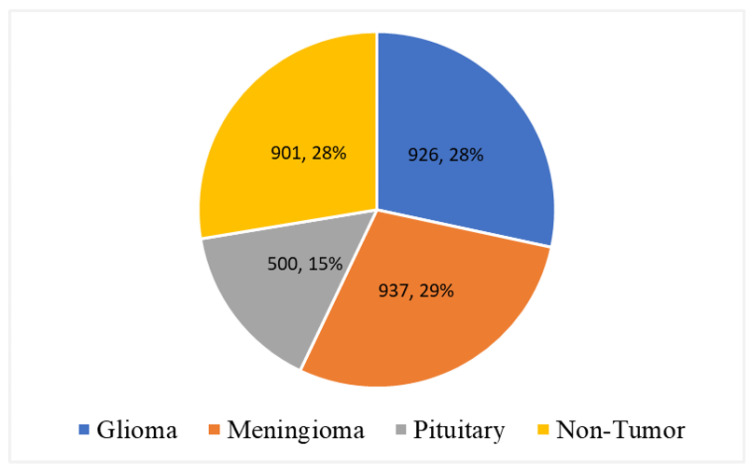
Data distribution analysis according to class labels.

Furthermore, Figure 7 shows data distribution analysis according to class labels. It found that 29% of data instances belong to class meningioma. Similarly, 926 (28%) and 500 (15%) MRI samples belong to glioma and pituitary, respectively. Furthermore, 29% of MRI samples belong to the class non-tumor.

#### 3.3.2. Pre-Processing of MRI Data

**Figure 8:**
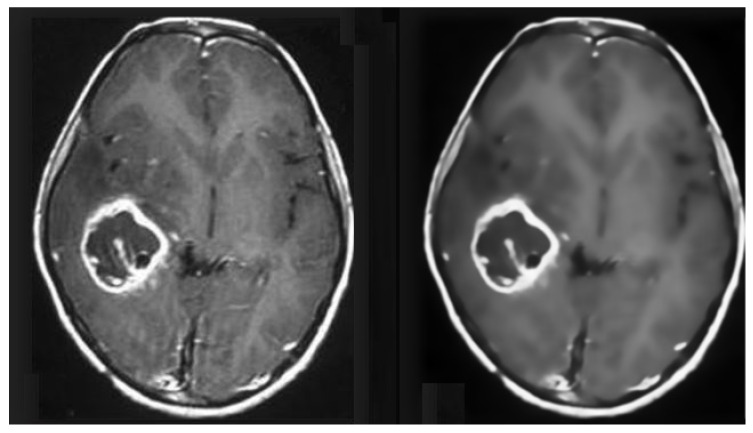
Pre-processed MRI data (Before and After Denoising).

MRI images suffer from Rician noise. The presence of noise can affect the accuracy of the classification model. Therefore, this noise needs to be removed before any classification can be performed in the pre-processing stage. For this purpose, an FSNLM filter was used. This technique uses fuzzy logic to find segments in the image that are homogeneous to the noisy pixel. Once these segments have been identified, they can be used to find the noise-less pixel. The FSNLM filter’s advantage over other denoising techniques is its low computational cost and its ability to retain the level of details in the MRIs. The FSNLM has been reported with a mean PSNR of 19.7 for T1 weighted images and 17.5 for T2 weighted images [22]. Figure 8 shows the actual noisy and denoised MRI.

#### 3.3.3. Proposed Multi-Scale CNN Architecture

This research study develops a CNN model based on a multi-scale architecture to detect and classify brain tumors from prepared MRIs. The CNN performs a convolution operation on the input images to extract promising features. However, the extracted features depend on the size of the kernel. A traditional approach uses only one kernel size. The size of the kernel restricts the features that can be extracted from the images. The multi-scale approach uses more than one kernel size, which allows the extraction of a wider range of features from the input images. Each parallel path extracts distinct features from the images. The output of each path is passed to the depth concatenation layer. The depth concatenation layer is deployed to concatenate the features map and pass them to the fully connected layer.

**Figure 9:**
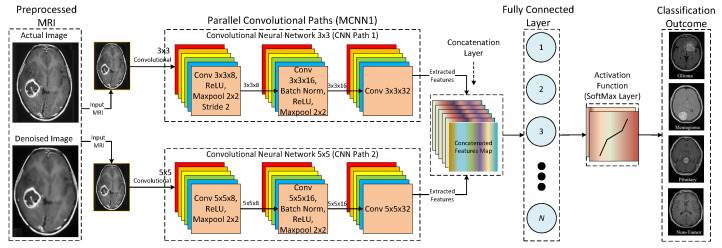
Detailed architecture of the proposed MCNN1.

Furthermore, three different multi-scale architectures are used for the proposed CNN model. These three architectures are named MCNN1, MCNN2, and MCNN3, and they have 2, 3, and 4 parallel convolutional paths, respectively. The reason for testing more than one multi-scale architecture is to understand the effect of adding more parallel paths to the performance. Figure 9 presents the detailed architecture of the proposed CNN (MCNN1) based on a multi-scale architecture with two parallel paths. The proposed MCNN1 consists of two parallel convolutional paths to extract features from the prepared MRIs. The same input image is passed to both parallel paths to extract different feature sets according to kernel size. Furthermore, it can be seen that two different kernel sizes are used, such as 3 × 3 and 5 × 5, to extract features and pass them to the concatenation layer for integration purposes. In addition, integrated features are flattened from a 2D matrix to a 1D vector and given to the fully-connected layer. Moreover, a fully-connected layer is an output layer that employs a Feed-Forward Neural Network (FFNN) to map inputs received from the previous layer into a classification outcome using a SoftMax method.

**Figure 10:**
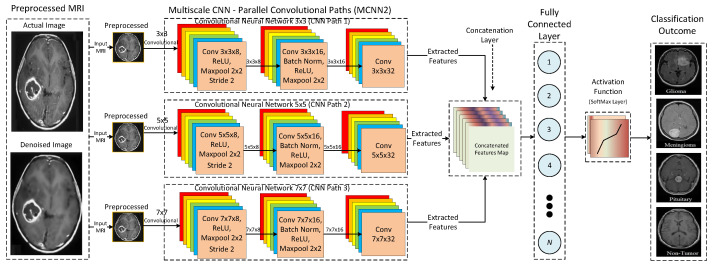
Detailed architecture of the proposed MCNN2.

Similarly, Figure 10 shows the detailed architecture of the proposed MCNN2 based on a multi-scale architecture with three parallel convolutional paths. The proposed MCNN2 uses three parallel convolutional paths with different kernel sizes, such as 3 × 3, 5 × 5, and 7 × 7, to extract rich features from the input MRIs. The variation in kernel sizes for each convolutional path extracts a distinct set of features. The reason behind using different kernel sizes for each convolutional path is to analyze the effect of multi-scale architecture in terms of accuracy and efficiency.

**Figure 11:**
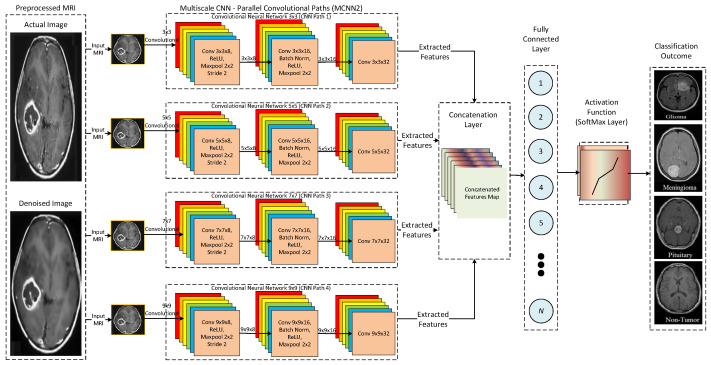
Detailed architecture of the proposed MCNN3.

Furthermore, Figure 11 presents a detailed architecture for the proposed MCNN3. In the MCNN3 model, parallel convolutional paths are increased to analyze the effect on the performance of the proposed multi-scale architecture. It uses four parallel convolutional paths with different kernel sizes, such as 3 × 3, 5 × 5, 7 × 7, and 9 × 9, to extract underlying features from input MRIs. Thus, three different multi-scale architectures are proposed to analyze the accuracy and efficiency of the proposed CNN model. Finally, the performance of the proposed CNN models is compared with state-of-the-art DL architectures, such as AlexNet and ResNet.

## 4. Experimental Results and Analysis

This section presents the experimental environment, evaluation metrics, experimental results, and analysis. First, an experimental environment of the proposed research study is presented. Second, different evaluation metrics are discussed to evaluate the performance of proposed and conventional DL architectures. Third, experimental results of proposed and state-of-the-art techniques such as AlexNet and Renset18 are discussed. Finally, comparative analyses show the impact of the proposed multi-scale CNN model over traditional CNN architectures.

### 4.1. Experimental Environment

The experiments are performed using Matlab R2020b on two MRI datasets: original and denoised images, to understand the effect of noise on the performance of the proposed multi-scale CNN models. Table 2 gives the specification of the systems on which all the experiments were performed. Furthermore, a dataset is divided into training and testing sample sets. The k-fold validation strategy is utilized to ensure the quality of the results. Moreover, the k-fold (i.e., k = 10) cross-validation method is used to ensure the generalization and fast convergence of the proposed multi-scale CNN models.

### 4.2. Evaluation Measures

For performance evaluation of the proposed model, four evaluation measures were used: accuracy, precision, recall, and F1-score [88]. These measures are calculated with the help of the confusion matrix. Table 3 shows an example of a confusion matrix for a binary classification problem.

Where $T_{g}$, $T_{m}$, $T_{p}$, and $T_{nt}$ indicate instances that were actually true and correctly predicted true; $T_{n}$ shows instances that were actually negative and correctly predicted negative. Similarly, $F_{n}$ indicates instances that were actually positive but falsely predicted negative; $F_{p}$ shows the instance that was actually negative but falsely predicted positive.

Based on the confusion matrix, different evaluation measures are constructed, such as accuracy, precision, recall, and F1-score.

Accuracy is defined as the ratio between the correctly predicted instances (true positive) and the total number of instances *N*. Accuracy is expressed for the aforementioned multi-classification problem as shown in Equation (5).

(5) $$\mathrm{Accuracy}=\frac{T_{g}+T_{m}+T_{p}+T_{nt}}{N}$$

Precision is defined as “out of all the actual positives, how many were correctly predicted as positive”. For multi-classification, it is defined for class label *i* as the sum over the *i*th row of the confusion matrix. Precision is expressed in Equation (6).

(6) $${\mathrm{Precision}}_{i}=\frac{M_{ii}}{\sum_{j}M_{ij}}$$

where *M* represents a matrix, *i* is defined for rows (predicted label), and *j* is defined for columns (actual label).

Similarly, recall is defined as “out of all the predicted positives, how many are actually positive”. For the multi-classification problem, it is calculated for the class label *i*th as the sum over columns of the confusion matrix. The recall is expressed in Equation (7).

(7) $${\mathrm{Recall}}_{i}=\frac{M_{ii}}{\sum_{j}M_{ji}}$$

Furthermore, the F1-score is defined as the harmonic mean of precision and recall. The F1-score is expressed in Equation (8).

(8) $$\mathrm{F1-score}=2*\frac{\mathrm{Precision}*\mathrm{Recall}}{\mathrm{Precision}+\mathrm{Recall}}$$

### 4.3. Experimental Results and Performance Analysis

This section presents the experimental results of the proposed and state-of-the-art DL architectures. In this research study, experiments were performed in four different stages.

- In the first stage, the raw images were used for classification by all models.
- In the second stage, the images were denoised using an FSNLM filter and were classified using all models.
- In the third stage, an artificial noise of magnitude 0.05 was added to the original images. These noisy images were then classified using all models.
- Finally, images with artificial noise were denoised using an FSNLM filter and sent to the classifiers.

#### 4.3.1. Original (Raw) MRI Data

In this stage, the proposed and existing DL architectures are employed on an original set of MRI data. Table 4 shows the accuracy, precision, recall (aka sensitivity), specificity, and F1-score of the five classification models on the original MRI dataset. The classification results indicate that the proposed MCNN2 model yields higher accuracy than other listed DL architectures. Furthermore, the classification results of the proposed MCNN2 model are significantly higher than the existing AlexNet architecture. Similarly, the classification performance of the proposed MCNN2 model is slightly higher compared to the ResNet architecture. Moreover, it shows that the proposed MCNN2 gives high specificity compared to other listed models. Based on the evaluation results, it is concluded that the proposed MCNN2 model gives an accurate classification rate on original MRI data compared to other DL architectures.

#### 4.3.2. Denoised Images Dataset

Next, Table 5 shows the classification results of the five classification models on the denoised images dataset. The classification results indicate that the proposed MCNN2 model gives superior performance compared to the AlexNet, ResNet, and other CNN architectures. Furthermore, the classification results of the proposed MCNN2 using denoised images are much better than the existing AlexNet architecture. It is also found that the performance of MCNN2 in terms of specificity is better than conventional CNN architectures. Similarly, it is also evident that the proposed MCNN2 model performed slightly better than the ResNet architecture. The evaluation results concluded that the proposed MCNN2 model gives high classification performance on denoised image data compared to other DL architectures.

#### 4.3.3. Synthetic Noise Based Dataset

Although some noise is present in the original images, the level of noise is very low. To deeply observe the effect on noise, a synthetic noise of 0.05 magnitude was added to the original images. These images were then classified using all five classifiers. Table 6 shows the classification results of all the classifiers on images with synthetic noise. The classification results using synthetic noise indicate that the proposed MCNN2 model performed significantly well compared to the AlexNet architecture. The resulting analysis shows that the proposed MCCN2 model improved accuracy by 6.73%, precision by 8.34%, recall by 5.44%, specificity by 7.65%, and F1-score by 6.91% compared to the AlexNet architecture. Furthermore, the classification results of the proposed MCNN2 were slightly better than the existing AlexNet architecture. The classification results using artificial noise data concluded that the proposed MCNN2 model gives high classification performance compared to the listed DL architectures.

#### 4.3.4. Synthetic Noise Removed

In the final stage, the images from dataset 3 are denoised using an FSNLM filter to remove the synthetic noise. Table 7 shows the classification results of these images. The evaluation analysis indicates that the MCNN2 gives higher accuracy, recall, and F1-score than AlexNet, ResNet, and other multi-scale CNN architectures. Furthermore, the ResNet architecture gives a high precision of 0.92301 compared to other DL architectures. Moreover, MCNN2 gives a slightly better specificity of 0.89671 than ResNet. Hence, our MCNN2 architecture yields high performance and outperforms state-of-the-art DL techniques.

## 5. Comparative Analysis

This section presents a comparative analysis to show the effectiveness and significance of the proposed research study. This research study proposes three different multi-scale architectures for brain MRI classification. The proposed architectures are named MCNN1, MCNN2, and MCNN3 and have 2, 3, and 4 parallel convolution paths, respectively. For performance evaluation, the proposed architectures’ classification results were compared with state-of-the-art CNN models, AlexNet and ResNet, using measures of accuracy, precision, recall, and F1-score. Accuracy was normalized between 0 and 1 to make it easier to present. The experiments were performed on four different datasets, each having a different level of noise.

**Figure 12:**
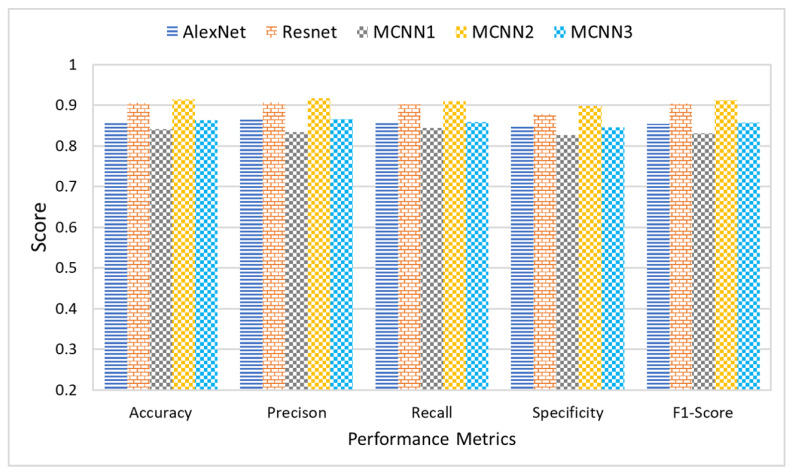
Comparative analysis of proposed and state-of-the-art DL techniques.

Figure 12 shows the average accuracy, precision, recall, and F1-score of the five classification models across the four different datasets. The comparison of the results indicated that the proposed architecture, MCNN2, performed better than state-of-the-art methods in all the evaluation measures. MCNN2 was able to classify the MRIs with an accuracy of 91.2% and an F1-score of 0.91%, which is higher than not only the other two proposed models but also the state-of-the-art techniques such as ResNet and AlexNet. Not only does the proposed model give better classification, but it does so at a lower computational cost. AlexNet and ResNet 18 have approximately 61 million and 11 million trainable parameters, respectively [89]; MCNN2 uses only around 1.8 million trainable parameters.

**Figure 13:**
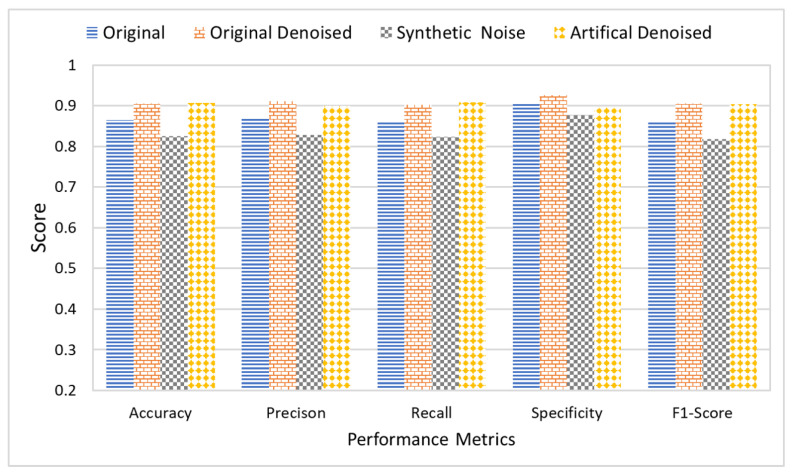
Comparative analysis of proposed and state-of-the-art DL techniques using noisy and denoised images.

Furthermore, the research study also studied the effect of noise on the classification results. For this purpose, all five classifiers were applied to the dataset in four different stages. Each stage had a different level of noise in the images. The original images with naturally existing noise were used in the first stage. In the second stage, images were denoised using an FSNLM filter. In the third stage, artificial Rician noise was added to the original image dataset. Finally, the images with artificial noise were denoised using an FSNLM filter in the fourth stage. Figure 13 shows the average classification results of all the classifiers on each of the datasets. The results indicate that the presence of noise adversely affects the classification results. While the original images produced decent results, they had some noise. However, when the images were denoised, it improved performance. Furthermore, adding artificial noise to the images resulted in a decline in performance of the classifiers. However, removing the noise from these images recovered the performance.

Moreover, Table 8 presents a comparative analysis to evaluate the efficiency of the proposed multi-scale approaches with state-of-the-art architectures, such as ResNet and AlexNet. The efficiency of the proposed and existing models is evaluated based on trainable parameters (weights and biases). The comparative analysis shows that our proposed multi-scale approaches require a small number of trainable parameters to extract features compared to ResNet and AlexNet. Our proposed multi-scale approaches drastically reduce the number of trainable parameters to extract features. The comparative analysis shows that the total number of trainable parameters for the proposed MCNN1, MCNN2, and NCNN3 is 3.443%, 8.191%, and 15.966% of the ResNet architecture. Similarly, it is also found that the total number of trainable parameters for the proposed MCNN1, MCNN2, and MCNN3 is 0.635%, 1.511%, and 2.946% of AlexNet. Hence, our proposed multi-scale approaches enhance efficiency for tumor detection in brain MRIs compared to the existing models such as ResNet and AlexNet [89].

## 6. Conclusions

The advancements in machine learning have paved the way for reliable computer-aided diagnosis systems, especially for MRI image classification. This study presents an effective and efficient deep learning model for brain tumor MRI classification. The model is compared with state-of-the-art techniques using accuracy, precision, recall, and F1-score. This research study presented a multi-scale CNN architecture for brain tumor MRI classification. The proposed model consisted of three parallel convolutional paths using kernel sizes of 3, 5, and 7. The model was tested on noisy and denoised images, and the results were compared with state-of-the-art CNN architectures: ResNet and AlexNet. The results portrayed that the proposed model MCNN2 not only gave better accuracy but also had less trainable parameters, decreasing its complexity. However, increasing the number of paths from three to four caused an immediate decline in the performance. The architecture with three parallel paths produced the best classification results. This study also analyzed the effect of Rician noise in MRIs on the classification performance of the deep learning model. The results indicated that the presence of noise in the MRIs caused a decline in the performance of the classification models; however, the effectiveness of FSNLM showed that MCNN2 can perform reasonably better once the image is denoised with FSNLM. The experimental results showed that increasing the filter size had a negative impact on the performance of the proposed architecture. Furthermore, the number of filters is empirically decided. The experiments are carried out without the supervision of a radiologist, and the findings are purely based on the dataset.

## Figures and Tables

**Table 1:** Data distribution as per class label.

#	Class	Number of Images
1	Glioma	926
2	Meningioma	937
3	Pituitary	500
4	Non-Tumor	901
5	Total	3264

**Table 2:** Implementation environment for the proposed multi-scale CNN architectures.

System Specifications	Description
Operating System	Windows 10
Processor	Intel Core i5, 2nd Generation
Main Memory	10 GB
IDE	Matlab R2020b
GPU	Nvidia GTX 750 TI

**Table 3:** Confusion matrix.

			Predicted Label		
		**Glioma**	**Meningioma**	**Pituitary**	**Non-Tumor**
Actual Label	Glioma	$T_{g}$	$F_{m}$	$F_{p}$	$F_{nt}$
	Meningioma	$F_{g}$	$T_{m}$	$F_{p}$	$F_{nt}$
	Pituitary	$F_{g}$	$F_{m}$	$T_{p}$	$F_{nt}$
	Non-Tumor	$F_{g}$	$F_{m}$	$F_{p}$	$T_{nt}$

**Table 4:** Classification results using original (raw) MRIs data.

Classifier	Accuracy	Precision	Recall	Specificity	F1-Score
AlexNet	0.84557	0.86206	0.84261	0.85731	0.83727
ResNet	0.90917	0.91205	0.90809	0.87874	0.90906
MCNN1	0.82718	0.82698	0.82965	0.83143	0.82073
**MCNN2**	**0.91132**	**0.914**	**0.91153**	**0.90798**	**0.9122**
MCNN3	0.8318	0.84052	0.81573	0.82369	0.82157

**Table 5:** Classification results using denoised image data.

Classifier	Accuracy	Precision	Recall	Specificity	F1-Score
AlexNet	0.87890	0.88986	0.87859	0.85421	0.88032
ResNet	0.91981	0.91876	0.9144	0.8979	0.91593
MCNN1	0.89266	0.89851	0.89154	0.8691	0.89405
**MCNN2**	**0.94190**	**0.94454**	**0.93741**	**0.92617**	**0.94057**
MCNN3	0.89672	0.90182	0.89237	0.88351	0.89488

**Table 6:** Classification results using added synthetic noise to image data.

Classifier	Accuracy	Precision	Recall	Specificity	F1-Score
AlexNet	0.8104	0.80913	0.81326	0.79461	0.80584
ResNet	0.87423	0.87223	**0.8712**	0.85278	0.86941
MCNN1	0.7454	0.75964	0.74978	0.72584	0.73574
MCNN2	**0.87768**	**0.89251**	0.86761	**0.87821**	**0.87493**
MCNN3	0.81651	0.80728	0.81864	0.79431	0.80437

**Table 7:** Classifications results for denoised images.

Classifier	Accuracy	Precision	Recall	Specificity	F1-Score
AlexNet	0.89297	0.89629	0.88986	0.87921	0.89283
ResNet	0.91743	**0.92301**	0.88172	0.89283	0.91920
MCNN1	0.89908	0.84848	0.90323	0.86991	0.875
MCNN2	**0.92355**	0.91929	**0.92351**	**0.89671**	**0.92122**
MCNN3	0.90520	0.91297	0.90758	0.87231	0.90905

**Table 8:** Comparative analysis of trainable parameters for proposed multi-scale and existing DL architectures.

#	Classifier	Trainable Parameters
1	MCNN1	396,356
2	MCNN2	942,948
3	MCNN3	1,837,956
4	ResNet	11,511,784
5	AlexNet	62,378,334

## Data Availability

The data presented in this study are available on request from the corresponding author.
